# Color‐Bar Bedsheet: Affordable Assistive Tool for Dix‐Hallpike Test in Primary Care

**DOI:** 10.1002/jgf2.70184

**Published:** 2026-10-09

**Authors:** Suwicha Kaewsiri Isaradisaikul, Pitchayanan Namung, Sanathorn Chowsilpa

**Affiliations:** ^1^ Department of Otolaryngology, Faculty of Medicine Chiang Mai University Chiang Mai Thailand

**Keywords:** appropriate position, Benign paroxysmal positional vertigo, Dix‐Hallpike test, low‐cost diagnostic tool, medical assistance, primary care

## Abstract

**Background:**

Benign paroxysmal positional vertigo (BPPV) is a common cause of vertigo encountered in general practice. The definitive diagnosis relies on the Dix‐Hallpike test (DHT), which remains underperformed in primary care due to limited training and unfamiliarity among general practitioners.

**Aim:**

To develop the color‐bar bedsheet as a simple visual positioning guide for DHT and demonstrate its usefulness.

**Design and Setting:**

After the color‐bar bedsheet was developed, a prospective within‐subject comparative design in healthy participants was conducted.

**Methods:**

Healthy adult subjects with 141–190 cm height were recruited. Each examiner performed the DHT with and without bedsheet. The proportion of the appropriate position and the satisfaction of subjects and examiners were compared.

**Results:**

Sixty subjects and 12 examiners were enrolled. Subjects' average age was 37.8 ± 15.0 years and average BMI was 22.5 ± 3.6 kg/m^2^. The DHT with the bedsheet showed significantly better appropriate positions when compared to the group without using the bedsheet (65% vs. 18.33%, *p* < 0.001), with the odds ratios (OR) of 8.27. Also, there was significantly higher satisfaction of subjects and examiners in DHT with the bedsheet, compared to DHT without the bedsheet (*p* < 0.001).

**Conclusions:**

The DHT with the color‐bar bedsheet provided an 8 times higher chance of appropriate position (particularly in individuals under 170 cm in height) with better satisfaction of both examiners and subjects. The bedsheet could be used as an assistive tool for the DHT, especially for novice examiners, in order to increase the diagnostic yield of BPPV in primary care settings.

## 1 Introduction

Benign paroxysmal positional vertigo (BPPV) is a common cause of vertigo [1, 2, 3]. Patients with BPPV usually present with repeating, brief episodes of positioning vertigo and a positive positioning test without other ear and neurologic symptoms. The most common subtype (around 85%–95%) is the posterior canal BPPV (PC‐BPPV) [3, 4]. The Dix‐Hallpike test (DHT) is the gold standard test for PC‐BPPV. This test was introduced as a clinical bedside maneuver to diagnose BPPV in 1952 by Dr. Margaret Ruth Dix and Dr. Charles Skinner Hallpike [5]. The diagnostic indices of the DHT showed 79.3% sensitivity, 75% specificity, 95.8% positive predictive value, and 33.3% negative predictive value [6]. In patients presenting with a typical history of BPPV, the clinicians should perform the DHT to confirm the diagnosis [3, 7].

Steps to perform standard DHT include [3, 7] (1) the patient sits upright on a bed with extended legs; (2) the patient turns the head 45 degrees toward the examiner, to the tested side; (3) the patient moves quickly from the sitting to supine position with approximately 20 degrees neck extension below the horizontal plane of the bed with the examiner's support; the examiner observes for nystagmus and vertigo attack; (4) the patient returns to the upright position while maintaining the same head position and eyes open. In the positive DHT, the canalith reposition procedure (CRP; e.g., Epley maneuver) is recommended treatment. The steps of DHT and CRP should be done by experienced or trained examiners [8].

During the DHT, the examiner also plays an important role to provide sufficient support to the patient's head over the bed edge with maintaining 20 degrees of neck extension. Generally, examiners estimate the sitting height (upper body height) of the patient to provide the proper sitting location on the bed which facilitates suitable neck extension. Although machine‐assisted positioning tests or repositioning maneuvers are now available and provide accurate head angle [9, 10, 11, 12, 13], standard DHT remains widely affordable and is regularly used in all clinics. Despite the high prevalence of BPPV, the standard DHT remains underperformed in primary care, due to limited training and unfamiliarity among general practitioners.

In order to provide less examiner's effort on supporting the patient's head and assist the novice examiner to estimate the proper position, the “color‐bar bedsheet” was developed as a seated position landmark to facilitate the proper neck extension during DHT. This study aimed to: (1) compare the proportion of the appropriate position; and (2) compare the satisfaction of the subjects and examiners with and without the use of the color‐bar bedsheet during the DHT.

## 2 Methods

The study was approved by the Research Ethics Committee, Faculty of Medicine, Chiang Mai University (CMU), Chiang Mai, Thailand (certificate of ethical approval: No. 483/2021, study code: ENT‐2564‐08463). The prospective study was done at the otolaryngology clinic, Maharaj Nakorn Chiang Mai Hospital, in the examination room routinely used for the DHT.

### 2.1 The CMU Color‐Bar Bedsheet Preparation and Materials

1. Measured the bed's cushion routinely used for the DHT (the cushion's size was 184 cm in length, 61 cm in width, and 9 cm in thickness.) to use for the tailor‐made bedsheet prototype.
2. Prepared five pieces of 10‐cm width fabric, each a different color, with length equal to the width of the bed.
3. Measured the distance from the head of the bed to place the 5 strips of the fabric (Figure 1). The 1st, 2nd, 3rd, 4th, and 5th strips were at a distance of 40, 50, 60, 70, and 80 cm from the head of the bed, respectively.
4. Sewed the five colored strips onto the bedsheet.
5. The bedsheet was tested by two neurotologists.

**FIGURE 1:**
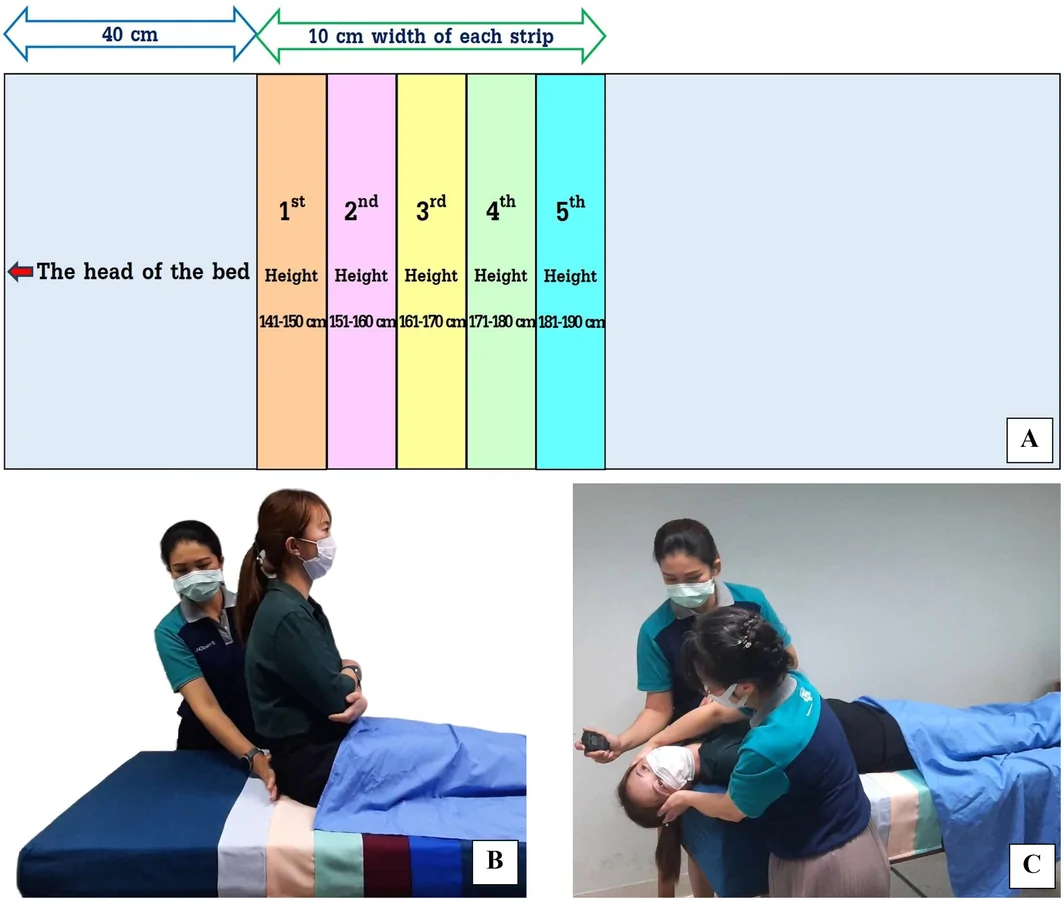
(A) CMU color‐bar bedsheet for assisting the Dix‐Hallpike test. (B) Example of the subject with a height of 151 cm is seated at the strip labeled 151–160 cm. (C) Supine position with a 45‐degree head turn and 20‐degree neck extension in the standard Dix‐Hallpike test for the right side. The assistant stands on the other side of the subject and holds the stopwatch in the examiner's view (a stopwatch is shown to illustrate its occasional use in ENT practice for documenting latency and duration of nystagmus, although this is not required in routine primary care settings).

### 2.2 Subject Recruitment

The examiners were residents in training at the Department of Otolaryngology, CMU, who had experience in performing DHT. Healthy subjects were recruited after the informed consent was done with the inclusion criteria as follows: aged 18 years or older, no postural hypotension or uncontrolled underlying disease, no limitation of neck or back, acceptance to complete all DHT steps, and height between 141 and 190 cm. The exclusion criteria were body mass index (BMI) 30 kg/m^2^ or above, diagnosed with cervical stenosis, Down's syndrome, severe rheumatoid arthritis, cervical radiculopathies, Paget's disease, morbid obesity, ankylosing spondylitis, low back dysfunction, retinal detachment, carotid stenosis or spinal cord injuries which are contraindicated for the DHT. Sample size calculation for two dependent proportions to achieve the first objective with 0.05 alpha and 0.20 beta was at least 57 subjects.

### 2.3 Testing Steps

The examiner performed the DHT twice; (1) DHT without the bedsheet, and (2) DHT with the use of the bedsheet, respectively. While using the bedsheet, the subjects were instructed to sit on the treatment bed according to the colored strip that corresponded to their height. for example, in Figure 1, the patient with a height of 151 cm is seated at the strip labeled 151–160 cm. The outcome measures were as follows; (1) the achievement of the appropriate position (defined by the patient's occiput lying on the bed edge with approximately 20 degrees of neck extension in a supine position, Figure 1); and (2) the satisfaction of the subjects and examiners (0 = least satisfaction and 10 = most satisfaction).

### 2.4 Statistical Analysis

The data were analyzed using Stata version 16.0 software (Stata Corp., College Station, USA). *p*‐value < 0.05 was considered as statistically significant.

## 3 Results

Sixty subjects (29 males; 48%, and 31 females; 52%), and 12 examiners (5 males; 42%, and 7 females; 58%) were enrolled in the study. Each examiner performed the DHT on five subjects. The demographic data of the subjects are shown in Table 1. The examiners' experience in performing the DHT was 2.67 ± 1.07 years on average (range 1–4 years).

**TABLE 1:** Demographic data of 60 subjects.

	Mean ± SD (range)
Age (years)	37.75 ± 14.98 (18–83)
Body weight (kg)	61.59 ± 13.91 (39–103)
Height (cm)	164.82 ± 12.29 (142–184)
Body mass index (kg/m^2^)	22.53 ± 3.59 (16.19–31.09)
Blood pressure in sitting position (systolic/diastolic; mmHg)	121.43 ± 9.62/76.7 ± 7.78 (108–140/62–94)
Blood pressure in supine position (systolic/diastolic; mmHg)	118.88 ± 9.30/74.88 ± 7.47 (98–140/60–92)

The DHT with the bedsheet assistance showed significantly better appropriate positions when compared to the group without using the bedsheet (65% vs. 18.33%, *p* < 0.001), with the odds ratios (OR) of 8.27 (Table 2).

**TABLE 2:** Proportion of appropriate position in the Dix‐Hallpike test with and without bedsheet.

	Appropriate position (%)	Inappropriate position (%)	OR (95% CI)	*p‐value*
DHT without bedsheet	11 (18.33)	49 (81.67)	Reference	< 0.001^a^
DHT with bedsheet	39 (65)	21 (35)	8.27 (3.99, 17.14)	

Abbreviation: OR, odds ratio.

^a^ Calculated by Generalized estimating equation using exchangeable correlation (GEE) model to adjust the data that was repeatedly measured in subjects.

The data were further categorized by the height of subjects, as shown in Table 3. With the use of bedsheet, the results showed significantly different proportions between the appropriate position and inappropriate position (*p* < 0.001). Additionally, larger proportions of appropriate position were observed in the subjects that did not exceed 170 cm in height.

**TABLE 3:** The proportion of appropriate position in the Dix‐Hallpike test with bedsheet, categorized by height of subjects.

Height (cm.)	DHT with bedsheet		*p‐value*
	Appropriate position (%)	Inappropriate position (%)	
141–150	11 (91.67)	1 (8.33)	< 0.001^a^
151–160	12 (92.31)	1 (7.69)	
161–170	11 (100)	0	
171–180	5 (33.33)	10 (66.67)	
181–190	0	9 (100)	

^a^ Calculated by Fisher's exact test.

With the use of bedsheet, there was a significantly better average satisfaction score in both subjects and examiners, compared to the DHT without using bedsheet (Table 4).

**TABLE 4:** Satisfaction scores of subjects and examiners while performing the Dix‐Hallpike test.

	Without bedsheet, mean ± SD (range)	With bedsheet, mean ± SD (range)	*p‐value*
Subjects (*n* = 60)	6.87 ± 1.02 (6.60–7.13)	8.97 ± 0.90 (8.73–9.20)	< 0.001^a^
Examiners (*n* = 12)	7.17 ± 0.90 (6.93–7.40)	9.32 ± 0.81 (9.11–9.53)	< 0.001^a^

^a^ Calculated by Pair *t*‐test.

## 4 Discussion

Increased diagnostic accuracy of BPPV leads to the selection of the correct maneuver and reduces unnecessary further diagnostic testing [14]. Performance variation during the DHT should affect the head position resulting in the diagnostic accuracy of the test. Our study performed the DHT according to its original description. A flat examination bed was used. The examiner stands at the subject's side of the tested ear, and the subject lies flat on a supine position with the head approximately 20 degrees extending over the bed's edge without a pillow. We developed the “color‐bar bedsheet” in order to improve the precision of the DHT during the neck extension that the subject's occiput should lie on the bed edge. This also assists the examiner in putting less effort into holding the patient's head. The results showed significantly better appropriate neck extension while using the bedsheet (65%) when compared to standard DHT without the bedsheet (18.33%).

To provide the exact head angle during DHT and CRP, the mechanical rotation chair was developed and claimed for better effectiveness [10, 11, 12, 13, 15, 16, 17, 18, 19]. The patient was seated in the chairs while the examiner controlled the position of the chair, resulting in more accurate head angles when compared to the traditional procedure. Although the mechanical rotation chair provides more standardization and has superior ability to a standard DHT, it is less available and much more expensive.

Despite the original DHT, several variations of the DHT were established and reported acceptable diagnostic accuracy. Jeon et al. [20] compared the modified DHT (using a 10‐cm thickness dense foam pillow under the shoulders) to the original DHT in BPPV patients. The diagnostic accuracy and the patients' discomfort were similar.

Andera et al. [14] presented a modification of the DHT termed the “loaded DHT”. The subject's head is flexed to 30 degrees in the plane of the affected PC and maintained for 30 s before placing the subject supine with a pillow under the shoulder. Three rounds each of standard DHT and the loaded DHT were performed in 28 patients. The severity score, duration of nystagmus, and sensitivity were higher in the loaded DHT than in the standard DHT.

Cohen [21] also proposed an alternative test, performed by brisk side‐lying with the nose turned 45 degrees away from the tested side. This test was designed for patients with limitations in range of motion. When compared to the original DHT, the slow‐phase eye velocity of nystagmus was similar.

Although the head angles were not exactly measured in each position of these modified diagnostic tests, these tests still provided good diagnostic accuracy when compared to the original version. A few studies reported that deviations of head angle during the Epley maneuver, in which the first two steps resembled the DHT, still provided successful treatment [22, 23]. This could be implied that slight deviations (not more than 20 degrees) [22, 23] of head angle still produced a response which was defined by positive nystagmus. However, the intensity and duration of nystagmus could be affected by other factors, for example, velocity and acceleration of head motion [24], fluid dynamics [24, 25], and characteristics of otolith particles (size, weight, initial position, and numbers) [24, 26].

Despite multiple factors affecting the diagnostic accuracy of DHT, the bedsheet still benefits novice examiners. As BPPV is a common cause of vertigo [1, 2, 3], the initial management is possibly done by general practitioners. The study by Ballve et al. [8] reported acceptable results of BPPV treatment by general practitioners in primary care who were trained by otolaryngologists. In our study, the percentage of the appropriate position with the use of the bedsheet was 65% and the chance of achieving the appropriate position was increased 8 times compared to the DHT without bedsheet. General practitioners who are not familiar with the procedure can use the bedsheet to provide the proper position during DHT.

Additionally, the Dix‐Hallpike test can be performed using two approaches: (1) the lateral approach, with the examiner positioned at the patient's side (as recommended in the clinical practice guideline [3] and used in this study), or (2) the cranial approach, with the examiner positioned behind the patient. Lieberz et al. [27] reported significant difference of head angles from the standard recommended angles during DHT, with the cranial tests showing significant deviations in extension and rotation angles, mean difference = 11.6° and 4.8°, respectively, while the lateral tests showing significant deviations only in extension angles (mean difference = 13.3°). However, the cranial test achieved the desired endpoints of 45° rotation more frequently, compared to the lateral tests (93% vs. 45%). Desired endpoints were defined as the minimum head angles required to adequately stimulate the posterior semicircular canal, consistent with standard diagnostic criteria. Figure 1 illustrated the lateral positioning, consistent with the standard DHT [3, 7]. Nevertheless, for general practitioners who may find the lateral approach less practical, the cranial approach can be considered as an alternative, offering potential ergonomic advantages for the examiner. Nowadays, both approaches are commonly used in clinical practice.

However, with the bedsheet, an increased proportion of inappropriate positions was observed in subjects heighted more than 170 cm. According to Table 3, the difference in subject height significantly affected the appropriate position. In our study, the bedsheet prototype was designed with five constant 10‐cm width strips for subjects' heights between 141 and 190 cm. Modification of this bedsheet prototype should be considered to improve the percentage of the appropriate position. Additionally, the efficacy of the bedsheet could be affected by sitting height (upper body height), which is probably different in individuals with similar total body height. The proper neck extension, which was defined by the occiput on the bed edge, could be varied due to the length of the neck and the BMI of individuals. Also, in our study, the head angles were estimated, not measured exactly.

Although our study demonstrated that bedsheet assistance significantly improved the accuracy of head positioning during the DHT (achieving an eightfold increase in correct alignment), there were several limitations. First, the fixed test order may have introduced performance bias, as examiners completed the second trial with prior experience. Future studies should employ a randomized crossover design with adequate washout periods to minimize this potential bias. Second, the prototype bedsheet was made from standard fabric, which may be susceptible to contamination. The future versions should consider other materials that could be cleaned easily with disinfectants, for example, plastic. Anatomical variations—such as sitting height, neck length, and BMI—could affect the efficacy of positioning, and head extension angles were visually estimated rather than precisely measured. Further studies should refine the design based on anthropometric data with adjustment the strip width related to the subjects' height and validate its use in patients with BPPV.

## 5 Conclusion

The use of a color‐bar bedsheet significantly improved correct positioning during the DHT—achieving an eightfold increase—and enhanced satisfaction among both subjects and examiners, particularly in individuals under 170 cm in height. The bedsheet assisted in approximating 20° of neck extension in the supine position and was especially helpful for novice examiners. However, further modifications are recommended to optimize alignment accuracy.

## Author Contributions

**Suwicha Kaewsiri Isaradisaikul:** conceptualization, data curation, funding acquisition, investigation, methodology, project administration, writing – original draft. **Pitchayanan Namung:** investigation, methodology, resources, writing – original draft. **Sanathorn Chowsilpa:** conceptualization, data curation, formal analysis, investigation, methodology, validation, writing – review and editing.

## Funding

This work was supported by Institutional Research, Chiang Mai University, Chiang Mai, Thailand (No. 61/2565 CMU MIS R000029641).

## Disclosure

This study was presented in an oral presentation session, at the International Congress of Otorhinolaryngology‐Head & Neck Surgery 2024 in conjunction with the 98th Annual Congress of Korean Society of Otorhinolaryngology‐Head & Neck Surgery, the 2024 Spring Meeting of Korean Association of Otorhinolaryngologists, held in Suwon, Korea from April 20 to 21, 2024 (abstract ID 980239).

## Ethics Statement

ENT‐2564‐08463 (Faculty of Medicine, Chiang Mai University).

## Conflicts of Interest

The authors declare no conflicts of interest.

## Data Availability

The data that support the findings of this study are available on request from the corresponding author. The data are not publicly available due to privacy or ethical restrictions.
